# Green Synthesis of Gallium-Based Metal-Organic Frameworks with Antibacterial Properties

**DOI:** 10.3390/molecules30214190

**Published:** 2025-10-27

**Authors:** Lin Teng, Yuxin Yang, Zhishang Shi, Yimeng Jia, Binbin Lu, Ying Zou, Shuo Liu, Libing Zhang

**Affiliations:** 1 School of Energy and Chemical Engineering, Tianjin Renai College, Tianjin 301636, China; teng_lin@tju.edu.cn (L.T.);; 2 College of Life Sciences, Nankai University, Tianjin 300071, China; 3 Tianjin Key Laboratory of Molecular Optoelectronic Sciences, Department of Chemistry, School of Science, Tianjin University, Tianjin 300072, China

**Keywords:** gallium, metal-organic framework, antibacterial, green synthesis

## Abstract

Bacterial drug resistance has become increasingly severe, with the development of novel antibiotics lagging far behind the evolution of resistant strains, drastically limiting clinical treatment options. Therefore, the development of new antibacterial materials is urgently needed. In this study, we synthesized a gallium-based metal–organic framework antibacterial material, designated as GM, with gallium as the central atom. Compared to a previously reported gallium-based MOF (FM), GM features a greener and milder synthesis process (room temperature, aqueous solvent, no toxic additives) while exhibiting improved antibacterial performance. Despite sharing identical raw materials, GM and FM are structurally distinct due to differences in synthesis methods, as evidenced by variations in morphology and crystal structure. Antibacterial assays against *Escherichia coli* (*E. coli*) and *Staphylococcus aureus* (*S. aureus*) demonstrated that GM outperforms FM, particularly against *S. aureus*, where GM exhibits threefold higher efficacy. Mechanistic investigations revealed that GM induces substantially higher intracellular reactive oxygen species levels and stronger disruption of bacterial membrane potential compared to FM, which may underpin its enhanced antibacterial activity. Additionally, cytotoxicity tests confirmed that GM shows no significant toxicity to mammalian cells. This study presents a gallium-based MOF prepared via a green synthesis route, with favorable antibacterial efficacy and biocompatibility, offering insights and a reference for the development of non-antibiotic antimicrobial agents.

## 1. Introduction

The alarming increase in microbial resistance has become one of the most serious health threats worldwide. The main reason for this is the improper and excessive use of antibiotics. Factors such as excessive prescriptions in clinical settings, the abuse of antibiotics in self-treatment, and the unrestrained use of antibiotics in agriculture and animal husbandry have accelerated the evolution of resistant pathogens [1,2,3,4]. These resistant microorganisms can evade the effects of conventional antibiotics, making infections that were once curable difficult to treat. Therefore, it is extremely necessary to explore and develop effective alternatives to traditional antibiotics [5,6].

Nanomaterials have emerged as a highly promising solution in the field of antibacterial applications, offering unique advantages over traditional antibacterial agents. Their nanoscale size endows them with distinctive physical and chemical properties, enabling them to possess various antibacterial mechanisms and being less likely to cause drug resistance [7,8,9,10]. For instance, metal and metal oxide nanoparticles (such as silver, zinc oxide, and titanium dioxide) can damage bacterial cell membranes, interfere with DNA replication, and produce reactive oxygen species to kill pathogens [11]. Carbon-based nanomaterials such as graphene oxide and carbon nanotubes exhibit strong antibacterial activity through physical penetration of cell walls and electrostatic interactions with microbial surfaces [12]. These materials have been successfully applied in various fields, including antibacterial coatings for medical devices, wound dressings with sustained antibacterial release functions, food packaging with extended shelf life, and water purification systems for eliminating harmful bacteria. These diverse applications highlight the potential of nanomaterials in addressing the crisis of antimicrobial resistance, namely, functioning as effective alternatives to antibiotics.

Metal–organic frameworks (MOFs) are a type of mixed crystal material composed of metal ions/clusters and organic ligands [13,14]. Recently, they have gained significant attention due to their use as novel antibacterial nanomaterials [15,16,17,18,19]. Their unique structural features, including extremely high porosity, large specific surface area, and tunable chemical functions, make them highly flexible in antibacterial applications. MOFs can serve as effective carriers for antibiotics, enzymes, or metal ions, enabling controlled and targeted release to enhance antibacterial efficacy while reducing side effects (such as biological toxicity). Additionally, certain MOFs exhibit intrinsic antibacterial activity through mechanisms such as generating reactive oxygen species, utilizing their porous structure to disrupt the integrity of bacterial cell membranes, or chelating essential metal ions from the microbial environment. These properties have encouraged the exploration of MOFs’ applications in various antibacterial scenarios, including surface coatings for medical implants, antibacterial textiles, and portable on-site disinfection systems, thereby further expanding the toolkit of solutions based on nanomaterials for combating antimicrobial resistance.

Gallium, particularly in its trivalent form (Ga^3+^), exhibits striking chemical similarity to iron(III) (Fe^3+^), a nutrient essential for nearly all bacterial pathogens. However, unlike iron, gallium cannot participate in redox reactions or support the catalytic functions required for bacterial metabolism. Due to its structural resemblance to Fe^3+^, Ga^3+^ is mistakenly taken up by bacteria, disrupts essential iron-dependent processes, leading to metabolic dysfunction even death [20,21,22,23]. As a result, gallium nitrate has been widely studied as an antibacterial drug and has been approved by the FDA [24,25,26]. While gallium nitrate has been approved for clinical use, several aspects still need to be addressed—particularly concerning its translation to and behavior within gallium-containing compounds. Given gallium’s potent antibacterial properties, the integration of gallium ions into MOF structures has become a focus of intensive research, combining the advantages of both components to enhance antimicrobial efficacy [27]. For example, Song et al. report the synthesis of gallium-based MOFs (Ga-MOFs) that exhibit significant antibacterial activity against *Porphyromonas gingivalis* (*P. gingivalis*), *Streptococcus mutans* (*S. mutans*), and *S. aureus*, with >90% bacterial killing at 1 μg/mL by disrupting biofilm formation, exopolysaccharide production, and bacterial membrane integrity [28]. Huang et al. used a similar method to prepare gallium-based MOFs, which were used as carriers for antibiotics (gentamicin) for antibacterial purposes [29]. They found that this material could enhance the drug’s penetration ability and improve the antibacterial effect of the drug. Wang et al. developed Ga/Cu-MOF nanozyme, which could be capable of loading antibiotics (vancomycin) and serve as a dual-metal ion reservoir for the sustained release of Ga^3+^ and Cu^2+^ [30]. The release of metal ions and antibiotics endows this MOF material with excellent antibacterial properties. Though excellent antibacterial ability, the above-mentioned materials are all obtained by traditional solvothermal synthesis, which has several critical limitations that hinder their practical translation: (1) Toxic organic solvents (*N*, *N*-dimethylformamide, DMF) and corrosive additives (trifluoroacetic acid) pose environmental risks and require complex post-purification; (2) high temperature (120 °C) and long reaction time (72 h) lead to high energy consumption. Our group synthesizes gallium-based MOFs loaded with antimicrobial peptide melittin (MM) at room temperature, which exhibit synergistic antibacterial activity against drug-resistant bacteria like *S. aureus* and *E. coli*, with good biocompatibility and the ability to accelerate wound healing [31]. Unlike the traditional solvothermal method [28,29,30], the preparation of MM is carried out at room temperature, using water as the solvent, and the reaction time is significantly shortened.

Over the past year, we have been focusing on the green synthesis of MOFs and exploring their applications in antibacterial fields [31,32]. Considering that the material preparation conditions in the reported reference were not environmentally friendly (using trifluoroacetic acid, DMF, and a reaction duration of 72 h at 120 °C), we aimed to develop a green synthesis route using water as the solvent and room temperature as the reaction condition using Ga nitrate hydrate and 1,3,5-benzenetricarboxylic acid (H_3_BTC) as raw materials. Our goal was not to reproduce FM via a greener method but to explore whether mild conditions could induce structural changes in Ga-MOFs and further enhance their antibacterial performance. This approach allowed us to evaluate both the environmental benefits of milder synthesis and the structure–function relationship of Ga-MOFs.

## 2. Results and Discussion

### 2.1. Preparation and Characterization of FM and GM

We synthesized gallium-based MOF according to the method described in Reference [28] and named it FM. Using the same raw materials and quantities, we replaced the reaction solvent with water and carried out the reaction at room temperature for an hour. Note that 1,3,5-benzenetricarboxylic acid is insoluble in water, and sodium hydroxide solution was added dropwise to make it dissolve. Under this condition, the addition of trifluoroacetic acid is not required. As a result, we synthesized another MOF and named it GM.

**Figure 1:**
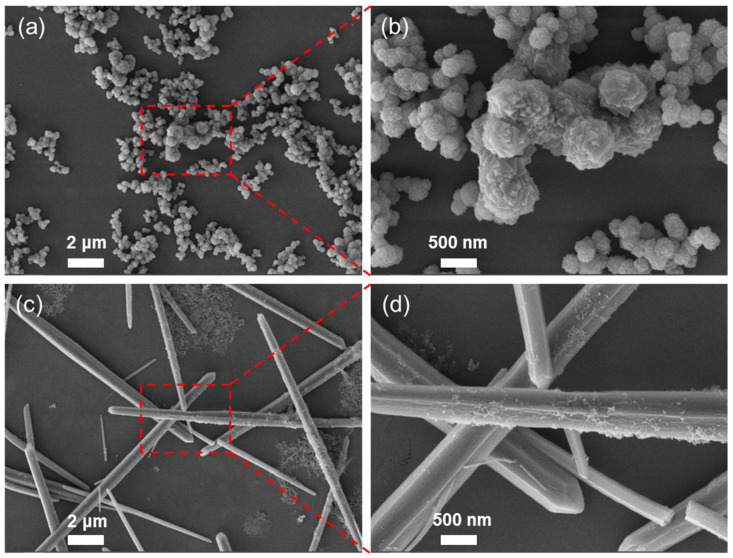
SEM images of FM (**a**,**b**) and GM (**c**,**d**) at different magnification.

The morphology of FM and GM was investigated using scanning electron microscope (SEM). Figure 1a,b show the morphologies of FM at different magnifications, which are consistent with those in reference [28]. The dimensions are approximately 400 nanometers, with a spherical shape and a spike-like outer surface. In contrast, GM is presented in a rod-like form, with a length of approximately several tens of micrometers and a width of around 500 nanometers (Figure 1c,d). The difference in morphology is associated with the change in synthesis conditions. For the synthesis of GM, NaOH adjusts the solution pH to ~8–9, ensuring complete deprotonation of H_3_BTC to BTC^3−^. Under neutral-to-weak alkaline conditions, BTC^3−^ prefers linear bridging coordination with Ga^3+^ (instead of branched coordination in acidic environments, e.g., trifluoroacetic acid used for FM), favoring 1D directional extension of the lattice. Just as in our previous research, using disodium terephthalate and gallium nitrate as raw materials and water as the solvent, the morphology of the obtained product (M0) was similar to that of GM [31]. This coordination behavior is also a well-documented trend across transition metal-BTC MOFs. For example, Sarkar et al. reported that pH = 8.2 (adjusted by triethylamine) induces complete deprotonation of H_3_BTC, enabling BTC^3−^ to form linear bridging coordination with Zn^2+^—a critical factor for synthesizing Zn-BTC MOFs [33]. Sahiner et al. demonstrated that at pH = 7 (adjusted by NaOH), H_3_BTC is fully deprotonated to BTC^3−^, which forms linear bridging coordination with Co(II)/Ni(II)/Cu(II) to generate regular morphologies (e.g., 1–10 μm square/rectangular Co(II)-Ni(II)-BTC MOFs) [34].

**Figure 2:**
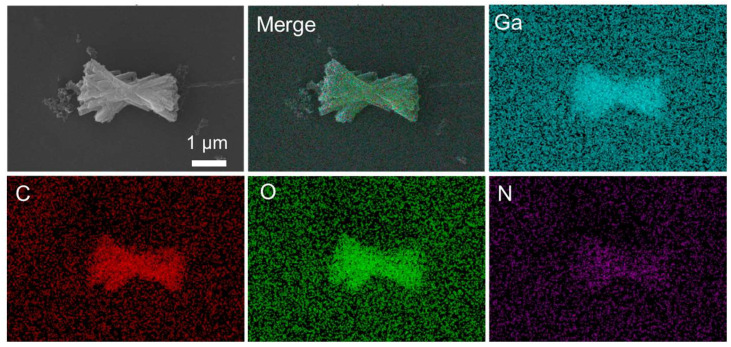
EDS mapping images of GM.

Energy dispersive spectroscopy (EDS) mapping experiments were then performed to analyze the elemental composition and distribution. As shown in Figure 2, Ga, C, and O distributed uniformly, with 14% mass percent of Ga. However, the signal of N element is obviously weak compared with other elements. Considering that the raw material contains nitrate ions, the low intensity of N element indicates that the synthesis of GM mainly comes from gallium ions and the interaction with BTC, rather than nitrate ions.

The results of the elemental analysis also confirmed the results of the EDS mapping. The percentage by mass of C, H, and N is 34%, 3.6%, and 0%, respectively, from the elemental analysis results. The raw material, gallium nitrate, consists of Ga^3+^ and NO_3_^−^, as there is no N element in GM, indicating that during the synthesis of GM, the NO_3_^−^ might not be incorporated into the framework structure, and they could be removed during post-synthesis treatment processes (washing).

**Figure 3:**
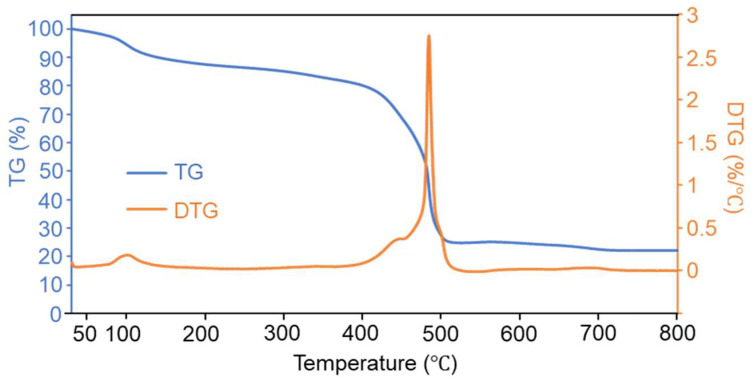
Thermogravimetric analysis results of GM.

The thermal stability of GM was then performed by thermal analyzer, with a heating rate of 10 °C/min from 30 °C to 800 °C in an air atmosphere. As shown in Figure 3, in the thermogravimetric (TG) curve (blue), GM initially shows a slight mass loss (about 10%) at lower temperatures (below 150 °C), which might be attributed to the removal of adsorbed water. Then, with the temperature increased to approximately 400 °C, the mass remains relatively stable, indicating that no significant thermal decomposition occurs in this temperature range. As the temperature rises beyond 400 °C, a rapid mass loss takes place, accounting for ~60% of total mass loss, which mainly attributed to the thermal degradation of the organic ligand BTC [34]. After this sharp mass loss, the residual mass tends to stabilize at higher temperatures (above 500 °C), representing the formation of thermally stable residues, most probably with the formation of Ga_2_O_3_. The derivative thermogravimetry (DTG) curve (orange) reflects the rate of mass change. A prominent peak appears at around 450–500 °C, corresponding to the rapid mass loss in the TG curve. This peak indicates the temperature range where the thermal decomposition rate is the highest. The small peak at around 100 °C corresponds to water loss. Above all, GM is relatively stable from room temperature to 400 °C.

**Figure 4:**
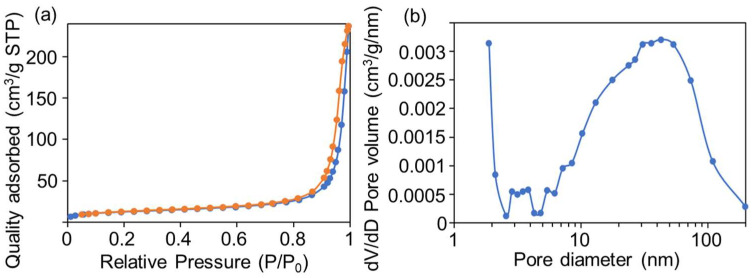
(**a**) N_2_ adsorption–desorption isotherm and (**b**) pore size distribution of GM.

The structural information related to porosity and surface area for GM was obtained by BET analysis. As shown in Figure 4a, nitrogen adsorption–desorption isotherms indicated that the surface area and pore volume of GM were 44.82 m^2^/g and 0.36 cm^3^/g, respectively. In the high relative pressure range (P/P_0_ > 0.8), the adsorbed amount increases significantly, which is consistent with the characteristic Type IV isotherm of mesoporous materials. Additionally, there is a hysteresis loop between the desorption curve and the adsorption curve, indicating that the material has a typical mesoporous structure. Overall, the pore structure and specific surface area characteristics of the material are in line with the common porous features of MOF materials [35,36]. From pore size distribution of Figure 4b, GM has a certain pore volume contribution in the small pore region (around 1–5 nm), but the pore volume is mainly concentrated in the mesoporous range (about 5–100 nm), indicating that GM’s pore structure is dominated by mesopores with a small amount of small pores, which is consistent with Figure 4a.

**Figure 5:**
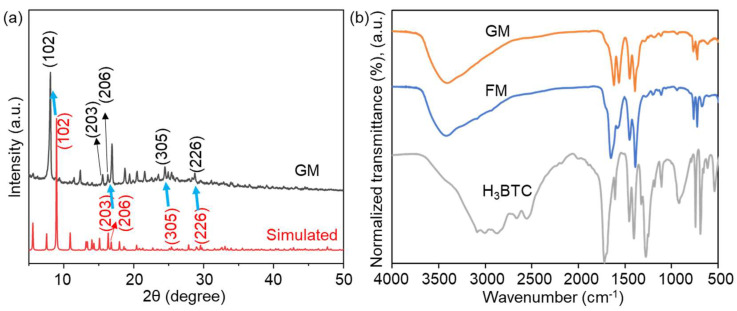
(**a**) XRD spectrum of GM and simulated pattern. (**b**) FT-IR analysis of GM, FM, and H_3_BTC.

The X-ray diffractometer (XRD) pattern in Figure 5a presents two curves: the gray curve corresponds to the experimental data of the sample, while the red curve represents the simulated diffraction pattern based on a theoretical crystallographic model. In the previous reference, the XRD peaks of FM were observed at significantly higher 2θ degrees compared to the simulated data (Figure S1). According to Bragg’s law (2d sinθ = nλ), this observation implies a reduction in interplanar spacing (d) of the crystallographic planes [28,29]. From Figure 5a we can see that all characteristic peaks match the simulated pattern, which confirms that GM retains the core crystallographic symmetry. However, the typical peaks of GM were observed at significantly lower 2θ degrees compared to the simulated data, and all the peaks can fit well with the simulated one. For example, the 2θ value of typical peaks (102), (203), (206), (305), and (226) for GM were all smaller than the simulated pattern (blue arrows in Figure 5a), suggesting that the characteristic peaks of GM have shifted in a general direction towards smaller 2θ values. Based on Bragg’s law, lower 2θ degrees means an increase in the interplanar spacing of its crystallographic planes. This expansion of d-spacing likely stems from lattice-level modifications, though the retention of labeled diffraction peaks (e.g., (102)) implies its core crystallographic symmetry remains unaltered. Considering the change in reaction condition, the change in XRD peaks for GM is rational.

Figure 5b shows the Fourier Transform Infrared Spectroscopy (FT-IR) analysis of GM, FM, and the raw material H_3_BTC. For FM, the strong absorption peaks at 1389/1450 and 1583 cm^−1^ suggested the Vsym and Vasym vibrations of the C–O group, respectively, revealing the introduction of H_3_BTC coordinated with the Ga atoms [29]. Similar absorption peaks at 1392/1450 and 1570 cm^−1^ were observed for GM. Moreover, the formation of -COOM characteristic peak at 1650 cm^−1^ (FM) and 1619 cm^−1^ (GM) illustrated the coordination interaction between metal and carboxyl group [37]. By comparison with the FT-IR spectrum of H_3_BTC, it can be seen that GM, like FM, successfully coordinates gallium ions (Ga^3+^) with H_3_BTC, proving the basic chemical structure of GM as a gallium-based MOF. Actually, minor differences in FT-IR peak intensities between GM and FM further support structural distinctions (e.g., differences in ligand coordination environment or lattice packing density), which complement XRD results to verify that synthesis conditions induce structural transformation.

These variations in XRD and FT-IR profiles, coupled with the morphological differences observed via SEM, collectively confirm that the green synthesis route (room-temperature, aqueous solvent) induced a structural transformation in the gallium-based MOF, distinct from the solvothermally synthesized FM. The differences in structure may confer different antibacterial capabilities to GM.

Figure S2 presents the ultraviolet-visible (UV–Vis) spectra comparison between freshly prepared GM (blue curve) and GM after 48 h of incubation in water (orange curve). Through the superposition analysis of the two curves, it can be observed that the spectral curve of GM after 24 h of incubation shows no significant differences from that of freshly prepared GM in terms of the position, shape, and absorption intensity of characteristic absorption peaks. This indicates that GM does not undergo obvious changes in its optical properties after 24 h of incubation in water, further proving that GM has good stability in an aqueous environment.

### 2.2. Antibacterial Activity of FM and GM

**Figure 6:**
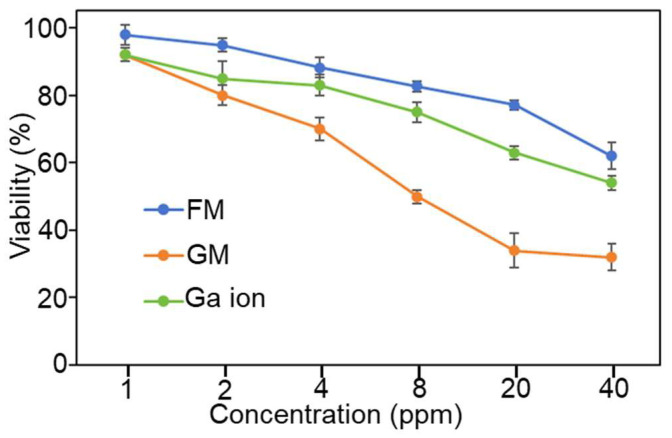
Antibacterial ability of FM, GM, and Ga ion on *E. coli*.

As mentioned earlier, FM has been proven to have antibacterial effects against *P. gingivalis, S. pyogenes,* and *S. aureus*. After we changed the preparation method, the GM obtained showed significant differences in characterization from the FM. Therefore, next we compared the antibacterial effects of the two materials, using *E. coli* and *S. aureus* as examples. As shown in Figure 6, three kinds of agents, i.e., FM, GM, and Ga ion, have been tested for the antibacterial ability of *E. coli*. It should be noted that there is no relevant research on the antibacterial effect of FM on *E. coli*. Our experimental results show that Ga ions have a certain antibacterial effect. At a concentration of 40 ppm, 54% of *E. coli* survived, which is consistent with the report in reference [31]. The antibacterial effect of FM is similar to that of Ga ions. In comparison, the antibacterial effect of GM is significantly higher than that of FM. At a concentration of 40 ppm, only 32% of the bacteria survive.

**Figure 7:**
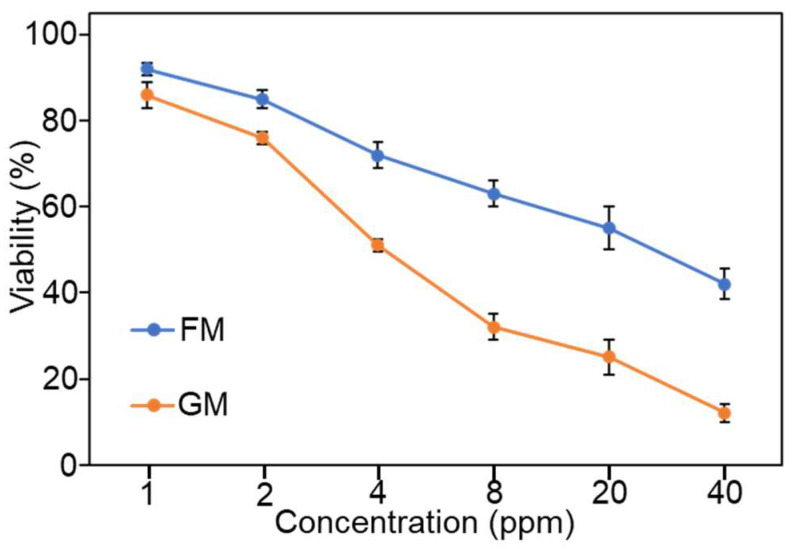
Antibacterial ability of FM and GM on *S. aureus*.

Next, we examined the antibacterial effects of FM and GM on *S. aureus* (Figure 7). It can be seen that the antibacterial effect of these two materials against *S. aureus* is significantly better than that against *E. coli*. With the presence of 40 ppm FM, the survival rate of bacteria was 42%. In the literature, when 1 ppm FM was present, the survival rate of *S. aureus* had already dropped below 20%. The reason for the inconsistency in the results is that the strains we used were clinical isolates, while the ones mentioned in the literature were standard strains [28]. On the other hand, the antibacterial experiments we conducted were not entirely consistent with the literature. Considering the differences in the used strains and experimental conditions, it can at least indicate that FM has a certain anti-*S. aureus* effect. Interestingly, under the same conditions, the GM we synthesized exhibited significantly better antibacterial effects than FM. At a concentration of 40 ppm, the bacterial survival rate was only 12%. Combined with the results of Figure 3, we modified the synthesis process of the material, making the reaction conditions more environmentally friendly. As a result, the antibacterial effect of the obtained material became even stronger. Although the antibacterial effect of GM is not as good as some previously reported materials, such as the MOF loaded with antibacterial peptides that we previously studied (achieving the same antibacterial effect at 5 ppm) [31], it should be noted that GM does not carry drugs and its antibacterial effect can still reach a common level in intrinsic antibacterial MOF [38,39].

### 2.3. Antibacterial Mechanisms

**Figure 8:**
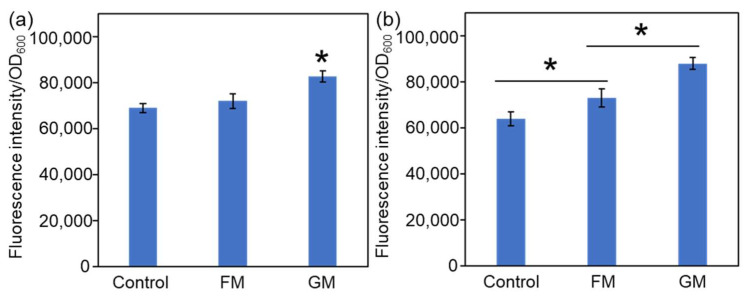
Changes in intracellular ROS levels following FM and GM treatment. (**a**) *E. coli*. (**b**) *S. aureus*. The asterisks (*) indicate significant differences between the groups (*p* < 0.05).

The antibacterial mechanisms of FM and GM were then studied. Reactive oxygen species (ROS) are triggered to eliminate pathogens when nanomaterials are taken up by pathogenic cells [40]. The effects of FM and GM on bacterial ROS production were then tested. As shown in Figure 8a, the presence of FM and GM can increase the ROS level in *E. coli*. However, the increase in ROS level in the FM treatment group is not very significant, while the GM treatment group can significantly elevate the ROS level. For *S. aureus*, the presence of FM and GM could significantly increase the ROS levels of the bacteria, and the ROS level in the GM treatment group was the highest (Figure 8b). The results in Figure 5 also indicate that the antibacterial efficacy of FM and GM against *S. aureus* is stronger than that against *E. coli*, and the antibacterial effect of GM is better than FM. As GM treated group exhibited much higher ROS level than FM treated group, potentially suggesting GM could induce higher level of ROS, which could effectively inhibit the bacterial growth.

**Figure 9:**
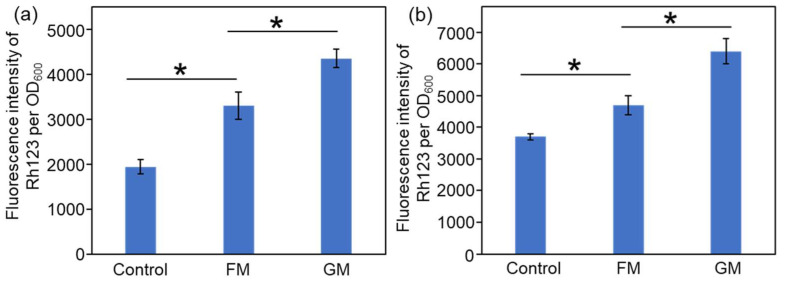
Bacterial membrane potential changes after FM and GM treatment. (**a**) *E. coli*. (**b**) *S. aureus*. The asterisks (*) indicate significant differences between the groups (*p* < 0.05).

Another common antibacterial mechanism is membrane depolarization [41]. The stability of the bacterial membrane potential is of great significance, as it relies on a steady electron transfer process across the membrane that generates a transmembrane proton gradient, which in turn supports the ATP synthesis essential for bacterial survival and function. The ability of FM and GM to disrupt the membrane of bacteria cells was then tested using Rh-123 as a fluorescent probe [36]. As shown in Figure 9, compared with control group with no treatment, the membrane potential after treated by FM or GM increased obviously, and the GM treated group exhibited the highest membrane potential.

Furthermore, there are reports in the literature indicating that rod-shaped materials may generate more ROS and are more capable of penetrating the cell wall. Therefore, they exhibit better antibacterial effects compared to particles with more uniform dimensions. Such as gold nanorods [42] and ZnO nanorods [43].

Consequently, compared with FM, GM exerted a stronger disruption effect on bacterial membrane potential, with higher ROS level, finally causing more cell death.

### 2.4. Cytotoxicity of GM

**Figure 10:**
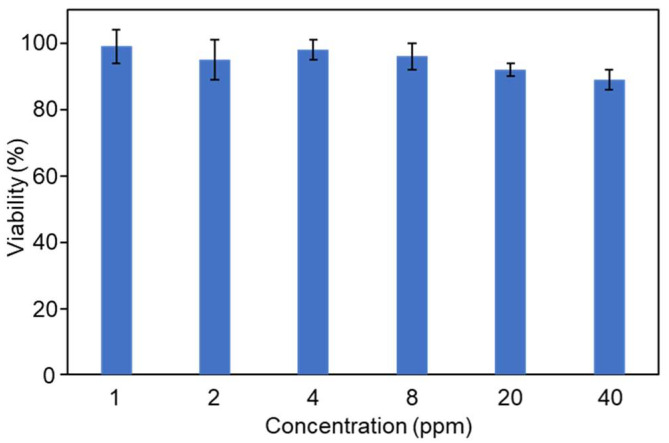
Cell viability of DC2.4 cells treated with GM for 24 h.

Considering the good antibacterial ability of GM, we also tested the cytotoxicity of GM towards mammalian cells. DC2.4 cells were used to test the cytotoxicity of GM using CCK-8 method [44]. As shown in Figure 10, at concentrations below 40 ppm, more than 90% of DC2.4 cells were alive after a 24 h incubation, indicating that GM showed no significant toxicity to the cells and has the potential for in vivo application.

## 3. Materials and Methods

### 3.1. Preparation of FM and GM

The synthesis method (solvent thermal method) of FM is detailed in reference [28].

For the synthesis of GM, Ga nitrate hydrate (100 mg) was first dissolved in 5 mL of water, which was set as Solution A. 1,3,5-benzenetricarboxylic acid (40 mg) was added in water (5 mL), NaOH (1 M) was added dropwise to make 1,3,5-benzenetricarboxylic acid fully dissolved (pH 7) and set as Solution B. Then, Solution A was added into Solution B dropwise under magnetic stirring. Then, the mixture was reacted for 1 h. After centrifugation (10,000 rpm, 2 min), white precipitation was obtained and washed three times by water and finally dried in vacuum at 60 °C overnight to obtain white powder.

### 3.2. Characterization of GM

The characterization of GM was mainly performed using a scanning electron microscope (SEM, TESCAN MIRA LMS, Brno City, Czech Republic), an X-ray diffractometer (XRD, SmartLab-SE, Tokyo, Japan), Fourier-transform infrared (FT-IR) spectrometer (ThermoFisher Scientific, Nicolet iS50, Waltham, MA, USA), an elemental analyzer (Elementar Vario EL Cube, Frankfurt, Germany), and a simultaneous thermal analyzer (HITACHI STA200, Tokyo, Japan). The stability of GM in water was tested by UV-Vis spectrophotometer (Persee, TU-1901, Beijing, China).

### 3.3. Bacterial Culture

*E. coli* and *S. aureus*, the representative Gram-negative and positive bacterium, respectively, were used for antibacterial test. Liquid Luria–Bertani (LB) medium (5 g/L yeast extract, 10 g/L tryptone and 10 g/L NaCl) was used to cultivate the bacteria. Typically, a single colony of bacteria was incubated in LB liquid medium at 37 °C with constant shaking overnight.

### 3.4. Growth Inhibition Assay

The concentration of bacteria suspension was first diluted to OD_600_ (optical density at 600 nm) = 0.02.

An amount of 100 μL of bacteria suspension was cultured in a 96-well plate and treated with different amounts of FM and GM, with a final volume of 200 μL in each well. The bacteria were then cultured at 37 °C for 24 h. The cells in each plate were counted using a microplate reader, and then the half inhibitory concentration (IC_50_) value was calculated.

### 3.5. Mitochondrial Membrane Potential Detection

A total of 100 μL of bacteria suspension (OD_600_ = 0.5) was cultured in a 96-well plate and treated with FM or FM (10 ppm). The bacteria were then cultured at 37 °C for 2 h, followed by incubation with 1 μM Rhodamine-123 for 3 h. After centrifugation, the cells were resuspended in PBS and analyzed using a microplate reader (excitation wavelength = 488 nm, emission wavelength = 520 nm).

### 3.6. Evaluation of Reactive Oxygen Species–Scavenging Activities

The antioxidant capacity of FM and GM was determined using the reactive oxygen species (ROS) kit (Solarbio, Beijing, China). A total of 100 μL of bacteria suspension (OD_600_ = 0.5) was cultured in a 96-well plate and treated with FM or GM (10 ppm). The bacteria were then culture at 37 °C for 2 h, followed by incubation with 1 µM 2′,7′-dichlorofluorescin diacetate (DCFH-DA) for 0.5 h. The fluorescence intensity of 520 nm was measured by a microplate reader (ex = 488 nm).

### 3.7. Cell Viability Test

DC2.4 cells were cultured in DMEM medium, including 10% (*v*/*v*) fetal bovine serum, 100 U mL^−1^ penicillin, and 100 U mL^−1^ streptomycin, in a humidified atmosphere of 5% CO_2_ at 37 °C. The cells were seeded in a 96-well plate and incubated for 24 h. Then, a series of GM concentrations (1, 2, 4, 8, 20, and 40 ppm) were added into the cell cultures, respectively, and the cells were incubated for another 24 h. Cell viability was detected by the CCK-8 assay kits (Solarbio, Beijing, China).

### 3.8. Statistical Analysis

Each experiment was performed in triplicate. The data were described as mean ± standard deviation (SD). All statistical analyses were performed using the ANOVA test (*p* < 0.05) using the SPSS software (Version 22, IBM, New York, NY, USA).

## 4. Conclusions

In summary, this study developed a novel gallium-based MOF (GM) through a green and mild synthesis approach, which operates at room temperature without organic solvents and exhibits superior antibacterial performance compared to the previously reported FM. Although GM shares the same raw materials as FM, the distinct synthesis conditions result in significant differences in morphology and crystal structure between the two materials. Antibacterial results revealed that GM exhibits markedly enhanced antibacterial activity, particularly against *S. aureus*, where GM at 40 ppm achieved over three times the bactericidal effect of FM at the same concentration. The superior performance of GM is attributed to its ability to induce higher intracellular ROS levels and more pronounced membrane potential disruption in bacterial cells compared to FM. Importantly, toxicity evaluations verified that GM retains potent antibacterial activity without significant cytotoxicity toward mammalian cells. These findings highlight GM as a promising eco-friendly, non-antibiotic antimicrobial agent, with potential applications in fields such as clinical therapy, medical devices, and food safety. Furthermore, this work provides a valuable reference for the green synthesis of functional MOFs and advances the development of alternative strategies to combat bacterial drug resistance.

## Data Availability

Data may be available to the corresponding author, Shuo Liu, upon request.

## Supplementary Materials

The following supporting information can be downloaded at: https://www.mdpi.com/article/10.3390/molecules30214190/s1, Figure S1: XRD spectrum of FM; Figure S2: UV–vis spectra of freshly prepared GM (blue curve) and after 24 incubation in water (orange curve).
